# Population genomics and connectivity of *Vazella pourtalesii* sponge grounds of the northwest Atlantic with conservation implications of deep sea vulnerable marine ecosystems

**DOI:** 10.1038/s41598-024-82462-z

**Published:** 2025-01-09

**Authors:** Anna Patova, Pedro A. Ribeiro, Francisco J. Murillo, Ana Riesgo, Sergi Taboada, Shirley A. Pomponi, Hans Tore Rapp, Ellen Kenchington, Joana R. Xavier

**Affiliations:** 1 Department of Biological Sciences, University of Bergen, Bergen, Norway; 2 Fisheries and Oceans Canada, Bedford Institute of Oceanography, Dartmouth, NS B2Y 4A2 Canada; 3 Departamento de Biodiversidad y Biología Evolutiva, Museo Nacional de Ciencias Naturales, Consejo Superior de Investigaciones Científicas, Calle de José Gutiérrez Abascal, Madrid, Spain; 4 Life Sciences Department, The Natural History Museum, Cromwell Road, London, SW7 5BD UK; 5 Departamento de Biodiversidad, Ecología y Evolución, Facultad de Ciencias, Universidad Complutense de Madrid, 28049 Madrid, Spain; 6 Marine Biodiversity Group, Departamento de Ciencias de la Vida, Universidad de Alcalá, 28871 Alcalá de Henares, Spain; 7 CIIMAR – Interdisciplinary Centre of Marine and Environmental Research of the University of Porto, 4450-208 Matosinhos, Portugal; 8 Harbor Branch Oceanographic Institute, Florida Atlantic University, Fort Pierce, FL 34946 USA

**Keywords:** Glass sponges, Genetic diversity, Hexactinellida, RADSeq, Migration, Bottom-fisheries, Sponge conservation areas, Conservation biology, Molecular ecology

## Abstract

Sponges are key ecosystem engineers that shape, structure and enhance the biodiversity of marine benthic communities globally. Sponge aggregations and reefs are recognized as vulnerable marine ecosystems (or VMEs) due to their susceptibility to damage from bottom-contact fishing gears. Ensuring their long-term sustainability, preservation, and ecosystem functions requires the implementation of sound scientific conservation tools. Here, the genetic diversity, structure, and connectivity of the deep-sea glass sponge, *Vazella pourtalesii* (Schmidt, 1870), was investigated using 1,102 neutral SNPs obtained in RADseq. This species is distributed across the northwest Atlantic from Florida, USA to Nova Scotia, Canada and we sequenced samples covering this full distribution and provided evidence of strong genetic structure with two distinct clusters: Florida together with the Carolina Shelves and the Scotian Shelf. We estimated moderate levels of diversity with low migration across large distances (> 1000 kms) and high connectivity at smaller scales (< 300 kms). Further, fishing pressure on genetic diversity was evaluated, within two Sponge Conservation Areas (SCAs) on the Scotian Shelf. Those areas have different disturbance histories, and cumulative fishing pressure. Slightly lower levels of genetic diversity were found inside the SCAs, and yet they encompassed a high proportion of the diversity observed within the Scotian Shelf. We provide baseline data for future monitoring of the SCAs, discussing our findings in the light of existing area-based management tools.

**Supplementary Information:**

The online version contains supplementary material available at 10.1038/s41598-024-82462-z.

## **Introduction**

Sponges (phylum Porifera) are essential ecosystem engineers of the marine realm which form complex three-dimensional habitats under particular environmental conditions^1–3^. Such habitats, known as sponge grounds, aggregations or reefs depending on their configuration, are characterised by a high density of single or multiple sponge species^4^. They can occupy vast extensions of the seafloor, from hundreds to thousands of km^2^, often representing a high proportion of the benthic biomass^5,6^. In the North Atlantic, these habitats typically occur along oceanic ridges, continental margins, and seamounts with the highest diversity and density in the bathyal zone, from 150 to 1,700 m depth^3,4^. At higher latitudes diverse multispecific communities are prevalent, dominated by tetractinellid (four pointed spicules) sponges (genera *Geodia*, *Stryphnus*, *Stelleta*) sometimes mixed with hexactinellid (six pointed spicules) sponges (genera *Schaudinnia*, *Scyphidium* and *Asconema*)^5–7^, while dense “monospecific” assemblages, as those formed by the glass sponges *Pheronema carpenteri* (Thomson, 1869), *Poliopogon amadou* Thomson, 1877 and *Vazella pourtalesii* (Schmidt, 1870), typically occur in more temperate areas at lower latitudes^8,9^. Sponge habitats provide important ecosystem services for the functioning of deep-sea benthic habitat worldwide^10^, and due to their enormous seawater processing capacity, they influence the hydrodynamics of the benthic boundary layer^11^. The process influences the circulation of essential suspended nutrients and particles, such as silica, nitrogen (nitrate and nitrite), potassium, carbon, and ammonium^12–14^, often scarce in the deep sea. Moreover, sponges and their remains (spicule mats and stalks) form firm substrate which is suitable for colonization and serves as habitat for other species, increasing the diversity of epibenthic macro-, and megafaunal communities^2,15^.

In recent decades, technological developments made it possible to advance resource exploitation into greater depths, making the deep sea a new frontier for bottom fisheries, mining, and hydrocarbons exploitation^16^. These human activities expose deep-sea species and habitats to chronic anthropogenic disturbance, direct and indirect effects of which might persist for extended periods^17–19^. On account of their vulnerability to such impacts, deep-sea sponge grounds were included in the list of threatened and declining species and habitats of the Oslo-Paris Convention for the Protection of the Marine Environment of the North Atlantic^20^, and meet the criteria for Vulnerable Marine Ecosystems (VMEs) as detailed in the guidance provided by the United Nations’ Food and Agriculture Organisation^21^.

The development and implementation of efficient area-based management tools such as marine protected areas (MPAs) or closure areas to protect deep-sea habitats (e.g., OECMs – Other Effective Area-Based Conservation Measures^22^), are particularly challenging, due to the inherent difficulties to survey these remote habitats, and often hindered by enormous gaps in knowledge of structuring species’ biology and ecology. The long-term survival of species, their adaptation to environmental changes, and their potential to recover from anthropogenic impacts depend greatly on their dispersal ability, the degree of connectivity between populations, and genetic diversity^23^. The dispersal of most benthic marine organisms occurs during early life stages through free-living larvae^24^. As the view of oceans as a “well-connected” system with great potential for long-distance dispersal is changing, it becomes clear that larval dispersal rates are highly variable across species and taxonomic groups^25,26^. Direct estimation of larval dispersal and connectivity for deep-sea species remains extremely challenging due to the limited access to study these organisms^27^ and little is known about larval duration and behaviour in sponges^28^. As such, indirect measurements using particle tracking biophysical transport models^29^ and genetic markers provide at present the best approaches to assess connectivity patterns^30,31^. Molecular methods, in particular, have provided considerable insight into the genetic diversity, structure and connectivity in marine sponges, allowing to estimate gene flow between populations and identify geographical and oceanographic barriers^32–36^. More recently, genomic approaches such as restriction site-associated DNA sequencing (RADseq) of Single Nucleotide Polymorphisms (SNPs) have successfully revealed fine-scale population structure^35,37–39^.

The glass sponge *Vazella pourtalesii* (Schmidt, 1870) is a habitat-forming glass sponge widely distributed along the eastern continental margin of North America, from the Florida and Carolina shelves and slope (USA) to the Scotian Shelf (Canada), where it forms dense and unique “monospecific” aggregations on its northern distribution range and have been observed at a depth between 87 and 498 m below sea level^8^. Presence of this habitat-forming species shapes community structure, and locally increases biodiversity and abundance of epibenthic megafauna and invertebrates on the Scotian Shelf^40,41^, while also playing a relevant role in carbon, nitrogen, and silicon cycling in the area^12,13^. However, this habitat has been negatively impacted by fisheries targeting benthic fish such as redfish and pollock for several decades, with sponge bycatch reported in thousands of kg^40^. Disturbance history may alter patterns of genetic diversity through selection and changes to demography^42^, and it is unknown whether the fishing disturbance on these sponge grounds has produced genetic changes in the populations of *V.pourtalesii*. Bottom fishing remains the greatest threat to the survivorship of the sponges as they are readily caught in the gear and do not survive the exposure to air that occurs on deck before they are discarded as the catch is sorted^40^. Other potential threats include storm-induced disturbances which episodically cause extreme turbidity persisting for several days^43^, although, *V.pourtalesii* is able to cope with elevated particle loads over such time scales^44^.

The uniqueness and rarity of this habitat alongside its fragility and sensitivity to bottom fishing warranted its classification as a VME by the Northwest Atlantic Fisheries Organization^45^. In 2013, *Vazella* grounds became partially protected under the Policy for Managing the Impact of Fishing on Sensitive Benthic Areas by the Government of Canada. Under this policy, two Sponge Conservation Areas (SCAs) – Emerald Basin and Sambro Bank SCAs – were established, covering a total of 259 km^2^ of the seafloor where the highest density of individuals was observed^40^. However, species distribution models^8,46^ showed that these closure areas represent just 1% of the *V. pourtalesii* predicted distribution on the Scotian Shelf. Assessing how well these closures are connected and how much of the species’ regional genetic diversity they protect is vital to ensure the sustainability of this habitat and the ecosystem services it provides.

Thus, our study aimed to investigate the genetic diversity, structure, and connectivity of the habitat-forming glass sponge *V. pourtalesii* in the northwest Atlantic, using a population genomics approach. Our objectives were to (i) investigate spatial patterns of genetic diversity and structure of *V. pourtalesii* both across its distribution range in the NW Atlantic and on the Scotian Shelf, we expect there to be significant isolation between the samples from the most southern sampled localities and the northern distribution since particle tracking did not show any connectivity^47^ (ii) estimate probable dispersal distances and connectivity between *Vazella* populations, (iii) assess genetic diversity in relation to disturbance history, inferred from records of cumulative fishing effort (assuming that fishing activities might have affected negatively genetic diversity), and (iv) assess genetic diversity levels in areas open *versus* closed to bottom fisheries on the Scotian Shelf to inform future monitoring, given the short timeframe since establishment of these SCAs. We compare our results with those from particle-tracking models developed for this species^47^ and discuss the implications of our findings in light of existing area-based management tools aimed at protecting these deep-sea vulnerable marine ecosystems.

## Materials and methods

### Sample collection, preservation, and DNA extraction

**Fig. 1:**
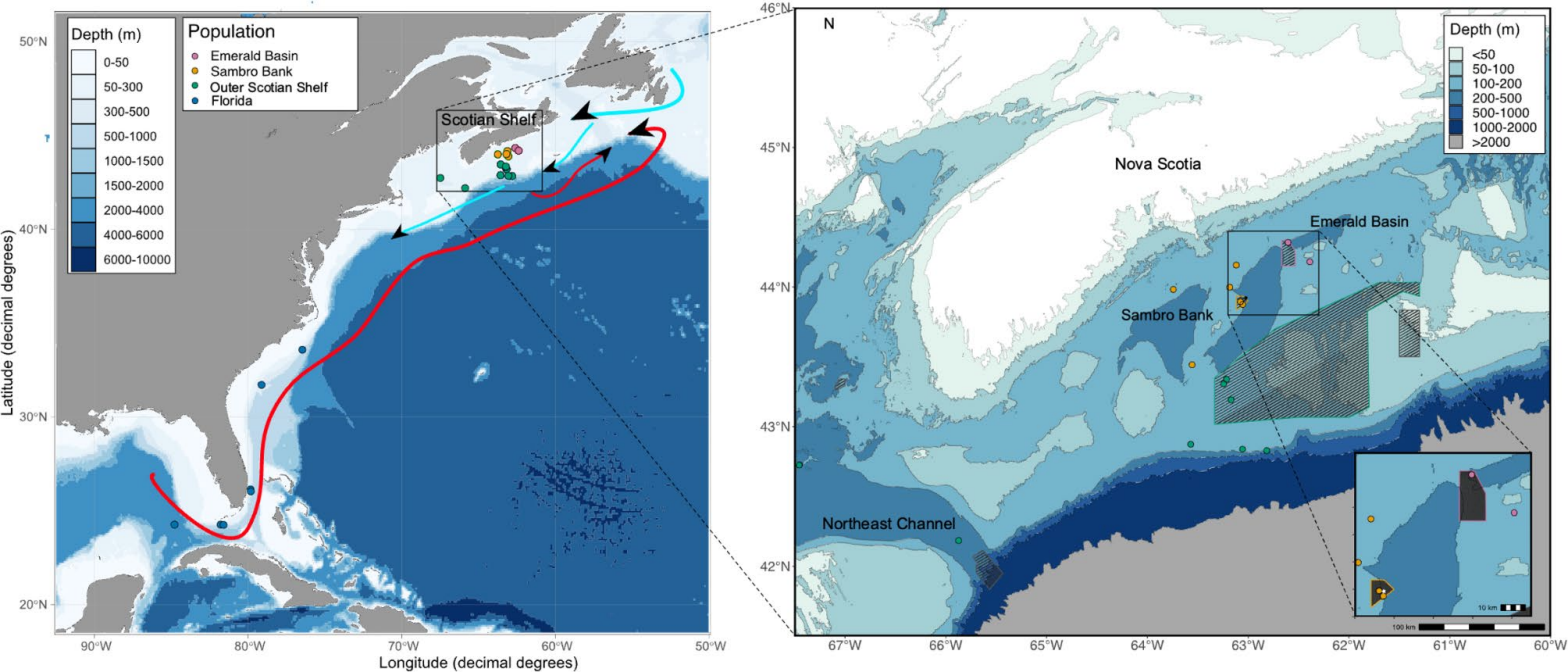
Map of the study area showing sampling sites of *Vazella pourtalesii* on the left panel the Scotian Shelf (Eastern Canada) and Carolina/Florida Shelves (Eastern USA). Arrows are a schematic representation of the Gulf Stream (red), and Labrador Current (blue). On the right panel more detail of the Scotian Shelf is shown, highlighting areas currently closed to bottom-contacting fisheries, including the two sponge conservation areas (Sambro Bank and Emerald Basin SCAs – filled polygons, stripped polygons – areas closed to bottom fisheries but not specifically dedicated to sponge ground conservation). The asterisk indicates the location of the three samples clustering in their own cluster with structure. Both maps were created in R^51^ using the ggplot2 library^52^, the conservation area shapefiles were obtained from (https://open.canada.ca/data/en/dataset/bb048082-bc05-4588-b4f0-492b1f1b8737/resource/ffc26f00-dfff-4fe8-a399-1ccffb6ce42d). The bathymetry data was obtained from (https://download.gebco.net, accessed 27th of July 2022). The maps were combined in Inkscape 1.3.2.

Specimens of *V. pourtalesii* were collected across the species distribution in the northwest Atlantic. Sampling sites spanned approximately 3,000 km across three ecoregions, the Scotian Shelf (eastern Canada), the Carolina Shelf, and along the continental margin of the Florida Shelf (the eastern parts of the southern USA), and depths ranging between 134 and 519 m (Fig. 1). Samples from the Scotian Shelf were obtained during four oceanographic campaigns: onboard the Canadian Coast Guard Ship (CCGS) *Hudson* in July/August 2016^48^, CCGS *Martha L. Black* in September 2017 (MLB2017001^49^), as well as CCGS *Alfred Needler* in August-September 2017 (NED2017020^50^), , and in August 2019 (NED2019030). These samples were collected using either the Remotely Operated Vehicle ROPOS (Canadian Scientific Submersible Facility, Victoria, Canada, MLB2017001), or a bottom trawl (NED2017020, NED2019030). Samples at the southern edge of distribution were obtained from the Harbor Branch Oceanographic Institute (HBOI) Marine Biotechnology Research Collection (MBRC), resulting from various scientific research campaigns through 2005–2010 (Table S1). Upon collection, small fragments of sponge tissue from each specimen were preserved in 96% ethanol and stored at -20^0^ C for subsequent processing.

Genomic DNA was extracted using the Qiagen DNeasy Blood and Tissue kit (Qiagen, Hilden, Germany). The manufacturer’s instructions were followed with the following minor modifications to increase DNA yield: cell lysis time was increased from 8 h to overnight (at least 16 h), RNase A (100 mg/ml) was added to remove residual RNA, and final elution was performed using 75 µl of elution buffer twice (150 µl total). DNA integrity was checked on a 1% agarose gel and was quantified with Qubit 2.0 double stranded DNA HS assay (Life Technologies). Extracts exhibiting high molecular weight bands, minimal smearing and high yield were selected for sequencing. A total of 98 samples (84 samples from the Scotian Shelf, and 14 samples from the Florida and Carolina Shelves) met the quality requirements and were used for library preparation. DNA concentration was normalized to 20 ng/µl.

### RAD-library preparation and sequencing

Library preparation and sequencing were performed at Floragenex Genome Sequencing and Analysis Facility (Portland, USA) on an Illumina HiSeq 2500 platform with 100 bp single-end reads in a high-density mode, following the protocol in^53^. Briefly, genomic DNA was digested with *PstI* restriction enzyme, followed by ligation of adapters (P1 and P2), Illumina sequencing primer sites, and barcodes. Each sample had a unique barcode (10 bp) for identification. The adapter-ligated fragments were subsequently pooled at equal concentrations into multiplexed libraries and randomly sheared. The fragments were size-selected using gel electrophoresis, and then index was attached to a second adapter (P2) followed by purification and elution in 50 µL with 5 µL of the product used in PCR amplification (18 cycles) and purified. The concentration of enriched libraries was checked for quantity using Qubit and qPCR prior to sequencing.

### Quality filtering and assembly of RAD-seq data

Program *process_radtags* included in the Stacks 2.0 package was used to demultiplex and quality filter raw reads^54^. RAD-tags were purified using a rescue option (-r), allowing a mismatch in barcode and RAD site sequence of no more than two bp. Reads with uncalled bases (-c) and low-quality scores (-q) were discarded. Sequencing of the 98 RAD libraries yielded 833.96 million reads (with an average of 8.5 million reads per sample), 13% of which were filtered due to low-quality base calling, absence of individual barcodes, or a RAD-cut site.

Sequence contamination from various putative sources were identified with Kraken2^55^ and discarded for downstream analysis. Reads identified to be off-target such as bacteria, archaea, viruses, and fungi ranged between 1.5 and 2.5% of the reads per sample.

De-novo assembly of RAD loci was performed with the software Stacks 2.0^54^ without a reference genome, with a preliminary sensitivity analysis for optimising the most critical parameters controlling this procedure, as suggested in^56^ using the default set of stacks parameters that maximized the number of loci that was covered in more that 80% of the individuals’. The final selected assembly parameters were: minimum stack (allele) depth of coverage (m = 3), the maximum number of mismatches allowed between stacks (alleles) within a locus (M = 2), and the maximum number of mismatches allowed between loci (*n* = 2), allowing gapped alignments. The catalogue was built from 30 samples selected to represent all groups (with high coverage and number of reads). In the end, a total of 725,557 loci were genotyped, with mean depth coverage depth 146.6X per individual (min = 42.3X and max = 625.6X). Eighteen samples that produced low numbers of reads and insufficient mean coverage (< 10X) were discarded, and therefore downstream analyses were performed on the remaining 80 samples.

The samples were grouped into four geographical areas with different spatial extents: three groups on the Scotian Shelf, referred to further as Emerald Basin, Sambro Bank, and the Outer Scotian Shelf, and one group representing the Florida and Carolina Shelves (Fig. 1).

The *populations* module of Stacks 2.0 was applied as a first filtering step with parameters: *r* = 0.2 (retaining SNPs which were present in at least 20% of the individuals), write-single-SNP (keeps only the first SNP of the RAD-tag to avoid linkage between SNPs), max-obs-het (maximum observed heterozygosity) set to 0.70 to remove paralogous sections of the genome merged into a single locus, and --min-maf (minimum allele frequency to process a locus) set to 0.05 to remove loci not informative at a population level as well as putative sequencing errors. The --p parameter was set to 2, which refers to the minimum number of populations a locus must be present to be processed. The resulting vcf files were further filtered using part of the *dDocent* pipeline^57,58^. In this step, we tested various filtering parameters to obtain the best SNP set with a maximum number of SNPs, taking into account the SNPs distribution across the dataset, geographical grouping, and minimizing the missing data. To visualize the effect of filtering on the proportion of missing data in the dataset a matrix condenser (graphical server) was used^59^ and Ward-clustering with Pearson’s Chi-squared tests, and also varying the amount of tolerance for the missing data per sample or per locus. In general, missing data and SNPs filtering did not have significant changes in STRUCTURE or DAPC results. The tests helped to increase confidence in the filtering decisions and to select the following filters: --max-missing 0.8 (to filter out genotypes called below 80% across all individuals), --mac 3 (to filter SNPs that have a minor allele count less than 3), --minDP 3 (minimum mean depth), and option --remove to exclude the listed individuals with high proportion of missing data.

The final dataset consisted of 1,206 SNPs genotyped at 80 individuals. The number of individuals successfully genotyped per area was: Emerald Basin = 16, Sambro Bank = 36, Outer Scotian Shelf = 21, Florida/Carolina Shelves = 7 (Table 1).

### Detecting SNPs under selection

Markers putatively under selection were identified using three different statistical approaches using the programs Bayescan Ver. 2.1^60^, LOSITAN^61^, and Arlequin 3.5.1.2^62^. Bayescan was run with the following parameters: 5,000 outputted iterations, 20 pilot-runs, 10,000 length of pilot runs, 100,000 burn-in, 10 thinning interval, 10 prior-odds suitable for our dataset size^60,63^. LOSITAN calculates FSTDIST2^64^ and was run with the following settings: 500,000 simulations, using options --neutral mean *F*_*ST*_ and --force mean *F*_*ST*_, --confidence interval (0.95), --false discovery rate (0.05), --mutation mode (infinite alleles). Arlequin was run under default parameters with 20,000 simulations. In total, 104 SNPs (8.6% of dataset) were identified as being under selection using Bayescan (6 SNPs), LOSITAN (57 SNPs; FDR = 0.05) and Arlequin (102 SNPs; *p* = 0.05) (Figures S1, S2, S3), and therefore 1,102 neutral SNPs were retained for downstream analyses. The SNPs identified as under selection were blasted against the NCBI database as well as *V. pourtalesii*’s draft transcriptome^65^, but no significant matches were found.

### Quality filtering and population structure analysis

Deviations from Hardy-Weinberg expectations were tested per population using the *perl* script “filter_hwe_by_pop.pl” form the *dDocent* pipeline, with the default minimum cut-off value for HWE p-value of 0.001. Loci were removed if they were out of HWE in more than one of the four populations for all downstream analyses.

Genetic diversity estimates (observed (H_o_) and expected heterozygosity (H_e_), nucleotide diversity (π), and inbreeding coefficient (F_IS_) were calculated using the *population* function in Stacks 2.0. Tajima’s *D* statistics were calculated per population using *vcftools* v0.1.16 (--TamajaD 1) to detect signs of demographic expansion^57^. Wright’s fixation index (*F*_*ST*_) was calculated using Arlequin and r-package *hierfstat* v0.5-7^66^, R version 4.0.1^51^ and tested for significance (*p* = 0.05) with 20,000 simulations.

Population structure was assessed using different approaches: STRUCTURE^67,68^, fineRADstructure v.0.3.2^69^ together with RADpainter v0.1^70^, *snapclust*^71^ & DAPC^72^ implemented in adegenet v2.1.2, and admixture v1.3.0^73^ which implement various approaches and statistics to assign the membership of each individual to a group.

STRUCTURE 2.3.4 uses a Markov chain Monte Carlo (MCMC) method to examine clusters (K), also known as Bayesian clustering algorithm. This approach estimates the proportion of ancestry an individual shares with populations and calculates the assignment probability of an individual to each cluster. We ran a MCMC simulation for 1,000,000 repetitions after a burn-in of 500,000 iterations. Putative K values were tested from 1 to 6 with a set of ten replicates per run. Structure Harvester^74^ was used to select the most likely number of clusters and average individual membership values were computed from the ten replicates of K values in CLUMPP 1.1.2^75^ with a non-greedy algorithm, running for 1,000 iterations. Results were then visualized in DISTRUCT 1.1^76^.

FineRADstructure creates a matrix of coancestry coefficients (without a priory information species for species identity or population) derived from a distribution of identical or nearest neighbour haplotypes among samples. We implemented a burn-in of 100,000 iterations, followed by 100,000 Markov chain Monte Carlo (MCMC) iterations, and generated the resulting tree under default parameters. R-scripts *fineradstructureplot.r* and *finestructurelibrary.r* were used to visualize results. Since this program performs better with linked loci, the population module in Stacks was used with 6505 SNPs, deselecting the option “write-single-SNP” and population groups, which allowed the inclusion of all linked SNPs in the different RAD-tags. This provides better resolution of population structure due to better assignment of nearest neighbour relationships through multiple SNPs rather than single biallelic which divides alleles at a locus into just two groups^69^.

We used discriminant analysis of principal components (DAPC) which graphically shows genetic divergence among populations with prior avoidance of group assignment based on sampling locations^72^. We ran two different modes: (i) with a pre-set number of groups (4) defined by geographic location, and (ii) with no specification of data clusters, finding an optimal number of groups by function *find.clusters* (function of *adegenet*) which uses successive K-means clustering, helping to avoid a “priory assignment” of individuals to groups. As this is a non-model-based method, it tries to maximize the differences between groups and at the same time keeps the minimal possible variation within groups. We set an optimal number of discriminant functions to retain according to the optimal scores from the cross-validation *xval-DAPC* function obtained from our data^72^.

To reveal population migrations patterns, the R package *DiveRsity* v1.9.5 was used^77^, implementing *divMigrate* function^78^, and calculating “D” (Jost’s D), “G_ST_” (Nei’s Gst), and N_M_^79^. All figures were drawn with ggplot2^52^.

### Fishing pressure on the Scotian Shelf

To assess the potential impact of bottom fishing on the genetic diversity of *V. pourtalesii*, we estimated the fishing effort of the mobile bottom-tending gears, such as trawls targeting groundfish and shrimp, in the areas where our samples were collected. Fishing effort, derived from fishing vessel monitoring systems (VMS) data within 1 × 1 km grid cells from the period 2005–2014, was extracted from a raster layer^80^ using QGIS 3.14.16 and used to assess which of the sampled locations experienced fishing impacts. Sponge samples falling into a given grid-cell were grouped based on whether they were in a protection area or outside, and if they had experienced fishing pressure or not. For each of the four groups, genetic diversity indices and inbreeding coefficient, calculated for each group using the same methods described above. The SCAs were implemented in 2009, banning all bottom contact fishing within their boundaries.

### Sampling permits

Sampling on the Scotian Shelf SCAs was performed with authoritative permission to Dr. Ellen Kenchington (DFO) from the Marine Planning and Conservation (MPC) section of Fisheries and Oceans Canada (DFO), Maritimes Region, after review of the sampling protocols for the Conservation Areas.

## Results

### Genetic diversity of *V. Pourtalesii*

Genetic diversity statistics of *V. pourtalesii* on the four areas considered, and on the whole dataset, are summarized in Table 1. The number of private alleles found in each area was very low, ranging from 0 in Emerald Basin to 4 in Carolina/Florida Shelves. Overall observed heterozygosity (*H*_*O*_) was moderate (0.213), ranging from 0.151 in Florida/Carolina to 0.256 in Emerald Basin. Overall expected heterozygosity (considered as genetic diversity) was also moderate (0.300) with the lowest values observed in Florida/Carolina (*H*_*E*_=0.264) and the highest in Outer Scotian Shelf and Sambro Bank samples (*H*_*E*_=0.319 and *H*_*E*_=0.320, respectively). Global inbreeding coefficient (*F*_IS_) was 0.271 and ranged from 0.132 in Emerald Basin to 0.373 in Florida/Carolina Shelves, indicating a tendency to non-random mating. Overall nucleotide diversity (π) was moderate (0.293), being lowest on the Florida/Carolina Shelves (0.259) and highest (0.315) in the Outer Scotian Shelf. Tajima’s D values were positive in all locations, ranging from 0.447 in Emerald Basin to 0.676 in Sambro Bank (overall value of 0.551), indicating balancing selection or sudden population contraction.

### Population structure and connectivity

**Fig. 2:**
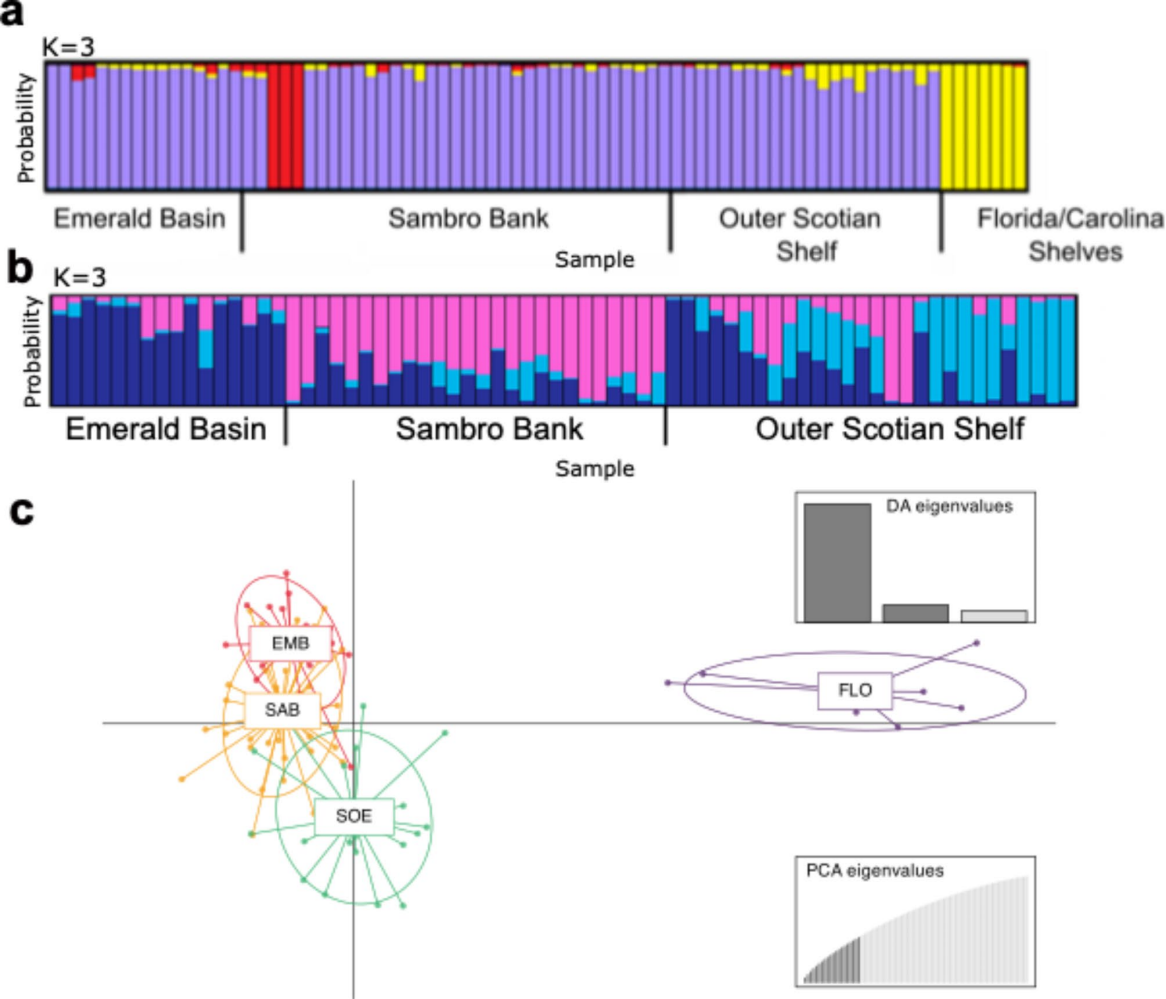
Genetic structure of *Vazella pourtalesii* inferred by STRUCTURE (**a**, **b**) Discriminant analysis of principal components (DAPC) (**c**), analysis of 1,102 neutral SNPs across the sampling area **a**) in the northwest Atlantic (*N* = 80, K = 3); and **b**) on the Scotian Shelf only, and excluding three individuals (SBB033, SBB034, and SBB035) of Sambro Bank (*N* = 70, K = 3). Each vertical bar represents an individual and the probability of its assignment to a particular genetic cluster (K) represented by different colours. Genetic structure using DAPC (**c**). The DAPC graph represents the individuals as dots and areas are abbreviated as follows: FCS as Florida-Carolina Shelves, EMB as Emerald Basin, SBB as Sambro Bank, OSS as the Outer Scotian Shelf.

STRUCTURE analysis on the neutral SNP dataset (1,102 SNPs), without location prior, suggested the presence of three clusters, based on the delta-K method (Fig. 2a). The first cluster (purple) consisted of Scotian Shelf samples (Sambro Bank, Emerald Basin, and the Outer Scotian Shelf), the second (red) included three samples from one location in Sambro Bank (SBB033, SBB034, and SBB035), and the third cluster (yellow) was formed by all samples from the Florida and Carolina Shelves (despite samples from these two shelves spanning > 1500 km); some individuals of the first cluster presented some degree of introgression from the other two genetic clusters (Fig. 2a). We also performed a hierarchical analysis excluding samples from the Florida and Carolina Shelves and three individuals from Sambro Bank red cluster (SBB033, SBB034, and SBB035) in order to identify possible substructure patterns in the Scotian Shelf. The optimal number of clusters inferred in the Scotian Shelf was also K = 3, although in this case admixture for some individuals was more prevalent than for the whole dataset (Fig. 2b). Most of the samples from Emerald Basin and a few samples from the Outer Scotian Shelf were assigned with a greater probability to the dark blue cluster, with admixture from the other genetic clusters > 30%. The majority of the samples from Sambro Bank and two samples from South Emerald Basin were assigned with a greater probability to the pink cluster, although some degree of admixture with the other two genetic clusters was also observed. As for the individuals from the Outer Scotian Shelf, apart from the samples mentioned before, some samples were assigned to a light blue genetic cluster, also with a varying degree of admixture from the other clusters. In other words, samples from the Outer Scotian Shelf appeared to have a mix of samples genetically similar to the ones from Emerald Basin and Sambro Bank together with samples distinct from these two areas (Fig. 2b).

However, upon visual inspection of the STRUCTURE output for this area, samples from the Emerald Basin are largely assigned to cluster blue-K1, those from Sambro Bank to pink-K2, and nine from the Outer Scotian Shelf to yet another cluster cyan-K3, several individuals have a mixed assignment probability to the different clusters, in various proportions. The graphical outputs for other K values can be found in (Figure S4). Importantly, in every tested scenario where K varied from K = 2 to K = 6 the Florida/Carolina Shelves samples were always found genetically distinct from all other areas and slight structuring on the Scotian Shelf was observed.

DAPC analysis without prior assignment of samples found the most optimal K = 1 using Bayesian Information Criterion (BIC), thus suggesting no separation of samples in genetic clusters. The subsequent DAPC analysis with prior assignment of samples to the various areas revealed largely similar p to the one in STRUCTURE analysis, with two main clusters clearly separating Florida/Carolina from the Scotian Shelf samples and further structuring on the latter, reflecting the different sampling areas. Similarly, considerable mixing between the Scotian Shelf areas, more prominent between Emerald Basin and Sambro Bank, and membership assignment to the different clusters was observed (Fig. 2c).

**Fig. 3:**
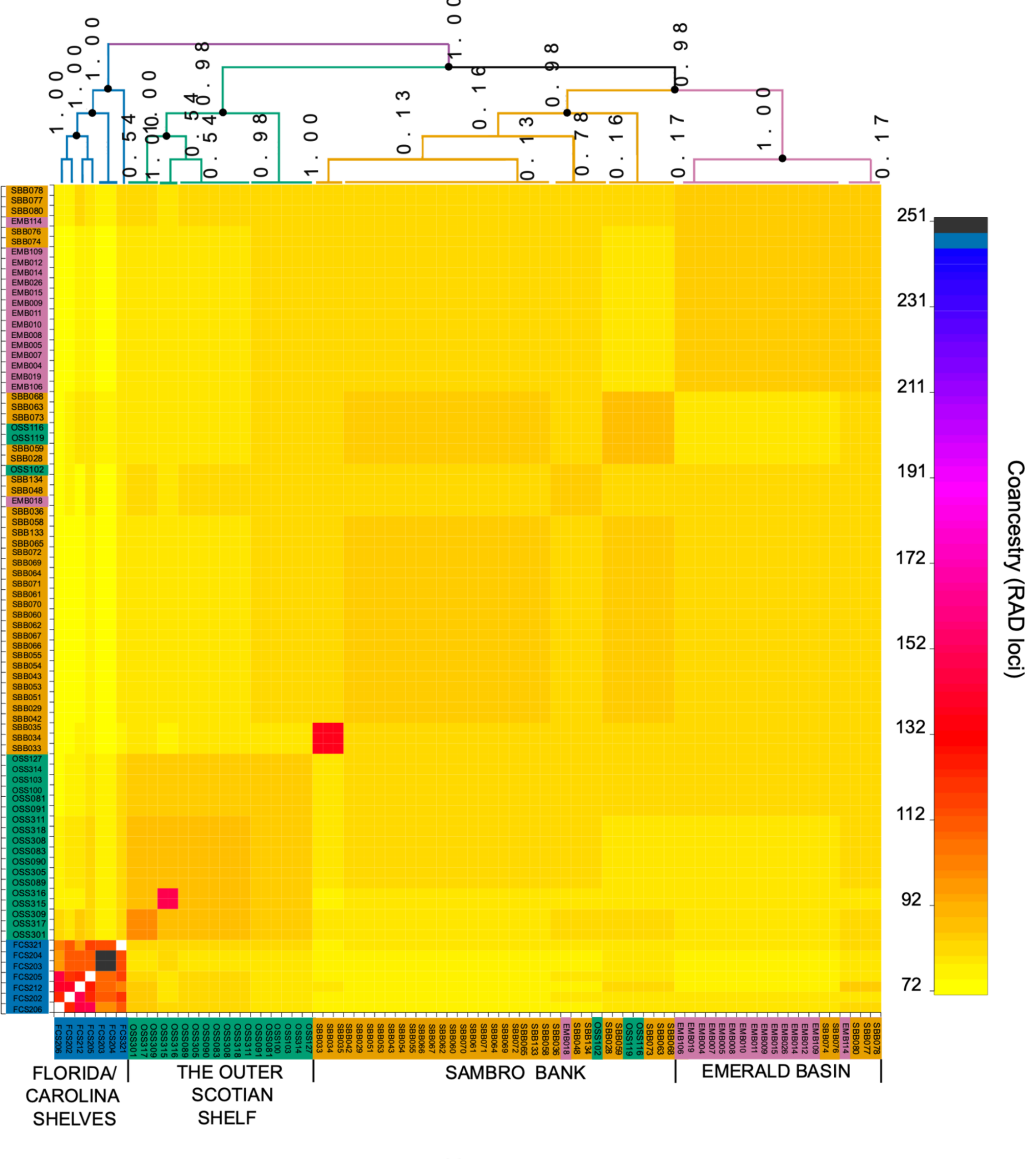
The heatmap represent a clustered coancestry matrix obtained from FineRADStructure analysis. The clustering follows the four sampled populations. The colours of the sample indicate the sampled population (blue = FLORIDA/CAROLINA SHELVES, green = OUTER Scotian Shelf, orange = SAMBRO BANK, purple = EMERALD BASIN). The samples from the Florida/Carolina shelves show a higher coancestry than the other populations. Weak but significant clusters of the other three populations are obtained, with some samples clustering outside of the sampled population. Overall, the coancestry is low and not indicating high levels of clonality.

The results obtained with the STRUCTURE and DAPC were further supported by the fineRADstructure analysis which revealed the clear presence of two distinct clusters with the Florida and Carolina Shelves cluster (blue) clearly separating from all Scotian Shelf areas with high support (PP = 1.0) (Fig. 3). The Scotian Shelf cluster further branched into two subclusters: the Outer Scotian Shelf (green), which only includes samples from this larger area (PP = 0.98); and a cluster largely composed of samples from Emerald Basin (purple) and Sambro Bank (orange) but that also includes three individuals from the Outer Scotian Shelf (OSS109, OSS116, OSS119) (PP = 0.98). The Sambro Bank-Emerald Basin subcluster is also branching into respective clusters with high support (PP = 0.98 and 1, respectively) but within these clusters there is more mixing of samples from the two areas. For example, four individuals (out of 36) from Sambro Bank (SBB074, SBB076, SBB077, SBB78) are grouping with the Emerald Basin, and three smaller clusters include individuals from more than one area, although these are unsupported (low PP values). Three individuals from Sambro Bank (SBB033, SBB034, and SBB035) and two individuals from the Outer Scotian Shelf (OSS315 and OSS316) show considerably higher co-ancestry between them than with other individuals of the same areas with which they also cluster. All individuals from Florida and Carolina Shelves also exhibit much higher co-ancestry between them in comparison to Scotian Shelf individuals. The fineRADstructure analysis also indicates that there is little to no clonal individuals in the dataset (Fig. 3).

All pairwise comparisons of genetic differentiation (*F*_*ST*_) were statistically significant at *p* < 0.05. Moderate pairwise *F*_*ST*_ values were found between the Florida/Carolina Shelves and the three areas within the Scotian Shelf, ranging from 0.129 to 0.158. Very low *F*_*ST*_ was found between the three Scotian Shelf areas (0.015–0.043) and global *F*_*ST*_ was also low (0.06) (Table 2).

**Table 1:** Population genetic statistics for *Vazella Pourtalesii* based on 1,102 neutral SNPs (variant sites only) across the sampling area in the Northwest Atlantic (*N* = 80) (upper panel), and on the Scotian Shelf only (*N* = 73), including within and outside the sponge conservation areas (SCAs). N – number of individuals; PA – private alleles; HE – expected heterozygosity; HO – observed heterozygosity; π – nucleotide diversity; FIS –inbreeding coefficient, D – Tajima’s D.

Sampling area	Fished	*N*	Depth (m)	PA	H_O_	H_E_	F_IS_	π	D
Geographic areas									
Emerald Basin		16	134–199	0	0.256	0.297	0.132	0.282	0.447
Sambro Bank		36	154–186	2	0.236	0.320	0.262	0.314	0.676
Outer Scotian Shelf		21	136–349	2	0.208	0.319	0.318	0.315	0.563
Florida-Carolina Shelves		7	281–519	4	0.151	0.264	0.373	0.259	0.516
Total		80	136–519	**–**	0.213	0.300	0.271	0.293	0.551
SCAs – Scotian Shelf									
Inside both SCAs		26	154–199	2	0.225	0.304	0.247	0.302	0.519
Emerald Basin SCA		*13*	199	0	0.246	0.293	0.145	0.291	0.384
Sambro Bank SCA		*13*	154–186	0	0.203	0.306	0.300	0.301	0.426
Outside SCAs		47	136–349	23	0.238	0.324	0.268	0.322	0.753
Total		73	136–349	**–**	0.232	0.314	0.258	0.312	0.812
Fishing pressure									
Inside SCAs	Yes, to 2009	26	154–199	2	0.225	0.304	0.247	0.302	0.519
Basin Closure	No	10	136–170	0	0.261	0.294	0.136	0.315	0.400
Outside SCAs and closures	Yes	30	133–237	4	0.241	0.318	0.259	0.325	0.660
Outside SCAs and closures	No	7	137–349	0	0.184	0.267	0.253	0.294	0.408

The italic numbers indicate subgroups of the 'Inside both SCA’.

**Fig. 4:**
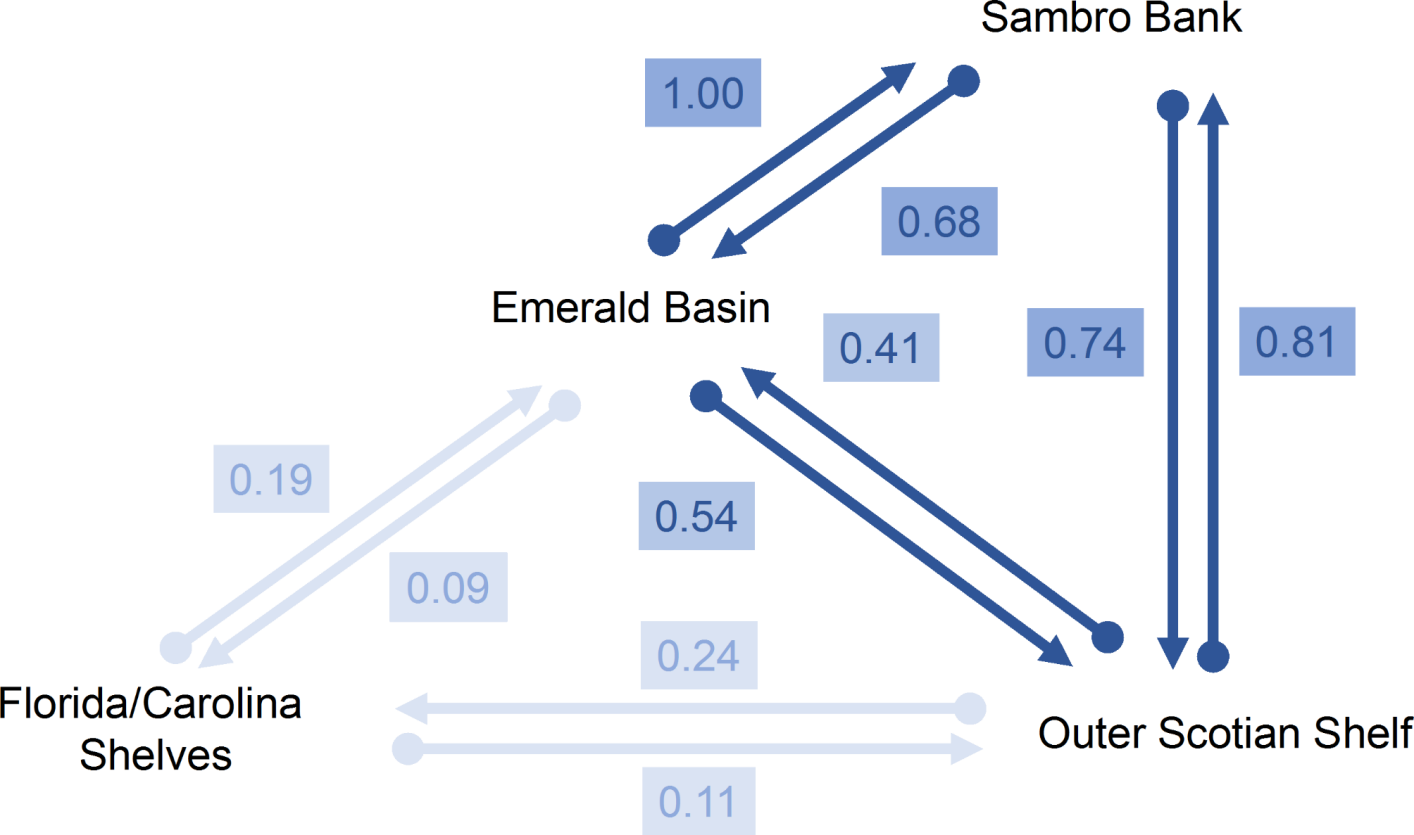
Directional relative migration (Nm, effective number of migrants) between *Vazella pourtalesii* populations inferred in divMigrate. The connections represent the two reciprocal migration components, and the shading the relative strength of migration between areas.

Migration analysis considering the four areas revealed varying levels of connectivity, inversely proportional with geographical distance between areas. The highest estimates of migration were found within the Scotian Shelf areas, i.e. at a scale of 300 km. These were lower between Emerald Basin and Outer Scotian Shelf (Nm of 0.41 and 0.54) than between any of these and Sambro Bank (Nm between 0.68 and 1). At greater distances, such as those separating the Scotian Shelf from Florida/Carolina (ca. 3000 km), migration significantly decreases (Nm between 0.09 and 0.27) and seems to occur mostly northwards, although no significant pattern of asymmetrical migration was detected in the analysis (Fig. 4). Additional measures of gene flow (Nei’s G_ST_, Jost D) showing similar patterns are presented in Table S2.

### Relationship between genetic diversity and bottom fishing pressure

**Fig. 5:**
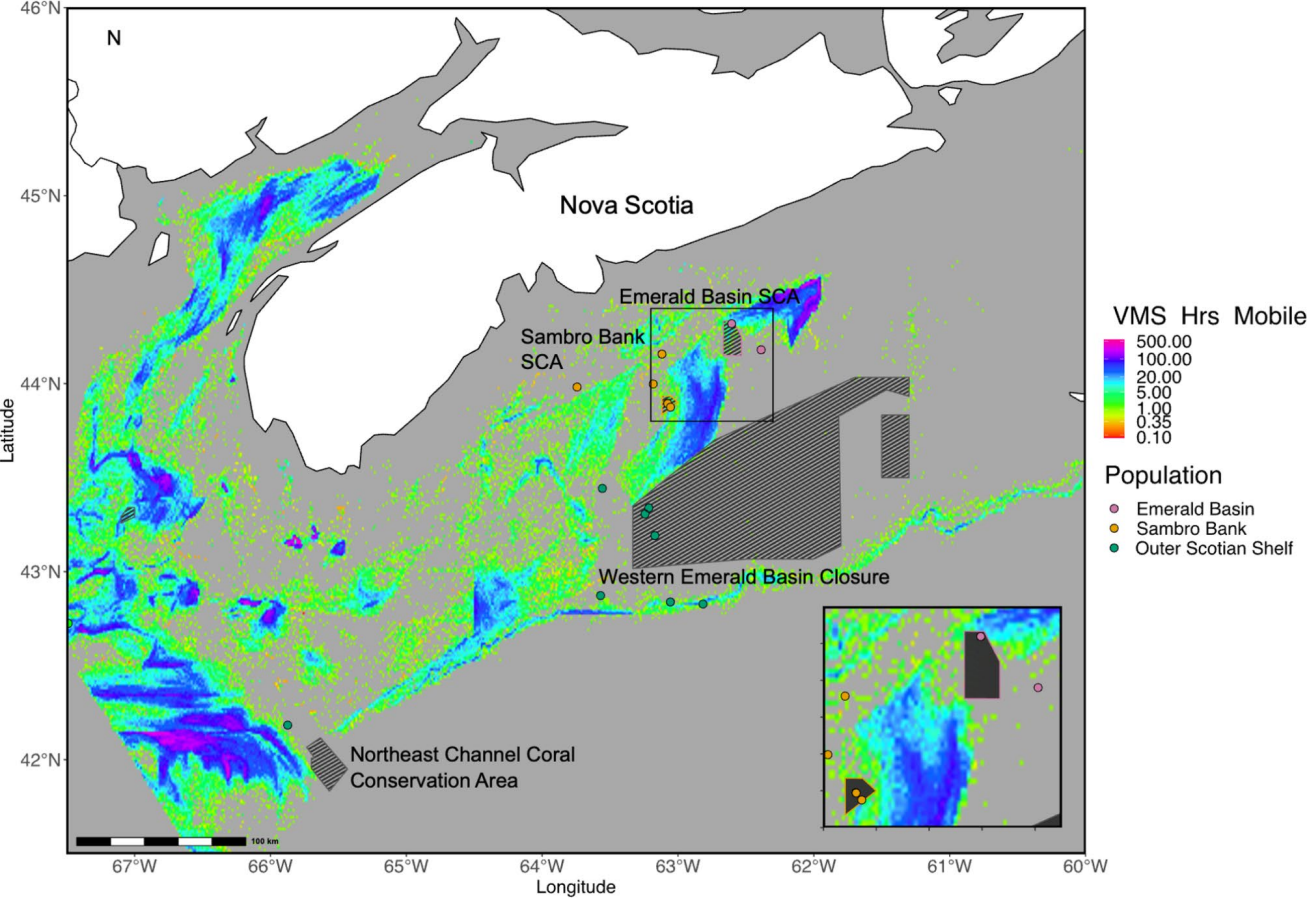
Distribution of the 2005–2014 fishing effort (hours recorded by VMS) on the Scotian Shelf for the groundfish mobile fisheries class (data from Koen-Alonso et al. 2018). Circles correspond to the sampling areas, filled polygons are the two Sponge Conservation Areas (Emerald Basin (purple) and Sambro Bank (orange)), and stripped polygons are the other areas currently closed to bottom fisheries. The map was created in R^51^ using the ggplot2 library^52 ^, the conservation area shapefiles were obtained from (https://open.canada.ca/data/en/dataset/bb048082-bc05-4588-b4f0-492b1f1b8737/resource/ffc26f00-dfff-4fe8-a399-1ccffb6ce42d). The VMS Hrs Mobile data was obtained from^80^.

Fishing effort of mobile bottom-tending gears in the period 2005–2014 was heterogeneously distributed across the Scotian Shelf, with higher effort located in areas in proximity to the protected areas (Fig. 5). The mean historical fishing effort in the locations (grid-cells), within the Sambro Bank and Emerald Basin SCAs was 10 times higher than in locations outside these closures from where the samples were analysed. This fishing effort was the highest within the Emerald Basin SCA (17.980 h), which is almost 14 times higher than in the surrounding area (1.33). A similar pattern was observed in Sambro Bank, where the calculated fishing effort (3.00) was more than twice the one observed outside the closures.

Comparisons of genetic diversity between SCAs, calculated separately for Sambro Bank and Emerald Basin and both closures combined *versus* the entire Scotian Shelf group, are presented in Table 1. Expected heterozygosity (*H*_*E*_) was marginally higher outside of the SCAs (0.324) than inside of them (0.304). The *H*_*E*_ was lowest for the Emerald Basin SCA (0.293) and the highest for the Scotian Shelf outside of closures (0.324). The number of private alleles outside of the closures (23) was much higher than inside the closures (2), with no private alleles unique to any of the SCAs. Similarly, both the allelic richness and overall nucleotide diversity was slightly higher outside than inside the SCAs. The inbreeding coefficient varied considerably between the closures, and was lower in Emerald basin (0.145) than in Sambro Bank (0.300). However, when both SCAs were combined, the inbreeding value (0.247) was only slightly lower than the values obtained for the entire Scotian Shelf (0.258) (Table 1).

**Table 2:** Pairwise population differentiation based on fixation index (FST) for the neutral SNPs dataset.

Sampling area	Emerald Basin	Sambro Bank	Outer Scotian Shelf	Florida/Carolina Shelves
Emerald Basin	–			
Sambro Bank	0.021*	–		
Outer Scotian Shelf	0.043*	0.015*	–	
Florida/Carolina Shelves	0.157*	0.129*	0.158*	–

*Significance level *p* ≤ 0.05.

Investing the genetic diversity statistics based on fishing pressure, we split the samples into four mutually exclusive groups. The lowest *H*_*E*_ and *H*_*O*_ values of 0.184 and 0.267, respectively were observed in the unfished areas outside closures,. The three other groups, had similar values ranging between 0.241 and 0.261 and 0.294–0.318, respectively. Inbreeding was lowest in the unfished Emerald Basin closure, with the others having much higher values (Table 1).

## Discussion

Glass sponges play important structural and functional roles in the deep-sea. The habitats they form (grounds and reefs) require close conservation attention to ensure their preservation from increasing pressures posed both by human activities and climate change^81^. Assessment of population connectivity and genetic diversity patterns provides valuable information for marine spatial planning, including the design and implementation of management and conservation tools (e.g., marine protected areas’ networks, fishing closures) in the deep-sea^82^. The *Vazella pourtalesii* sponge grounds on the Scotian Shelf have been protected from bottom-contact fishing through Canada’s establishment of two marine refuges (Emerald Basin and Sambro Bank Sponge Conservation Areas). They are considered to be ‘Other effective area-based conservation measures’ contributing to Canada’s marine conservation target of protecting 25% of its marine environment by 2025. The Convention on Biological Diversity (CBD) recognizes connectivity as an important element of creating ecologically sustainable networks of closed areas and OECMs are expected to complement MPA networks through improved connectivity amongst other attributes^22^. Our results provide such data for the important habitat-forming species, suggesting that *Vazella pourtalesii* forms two distinct populations in the northwest Atlantic with a limited connection between them and moderate levels of genetic diversity, providing important baseline data for future management of deep-sea conservation areas in the region.

Our study showed moderate levels of genetic structure among *V. pourtalesii* populations distributed along 3,000 km of the eastern continental shelf of North America. There was in particular a clear separation between individuals from the Scotian Shelf and those from the Carolina and Florida Shelves, whereas within these two regions genetic structure was, in comparison, less prominent. This implies that Canada and the United States of America should pursue independent but combined conservation actions for this species and not rely on trans-border subsidies.

Our findings detected gene flow among *V. pourtalesii* spanning all sampled sites, a common feature in deep-water sponges and other marine invertebrates inhabiting a similar bathymetric range^31,39^. Reproductive studies in other hexactinellid species – *Aphrocallistes vastus*, *Oopsacas minuta*, *Vitrolulla fertilis* and *Farrea sollasii* – suggest that *V. pourtalesii* may be a hermaphroditic and viviparous species with pelagic larvae able to remain in the water column for less than an hour to up to two weeks^3,28,83^. The ability to reproduce asexually, for instance through budding and bi-partition, has been reported for demosponges and glass sponges, including several rossellid species from the Southern Ocean^84^, and is a widespread feature among early metazoan lineages^85^, including corals^86,87^, bryozoans^88^ and sea anemones^89^. Asexual reproduction could thus influence the structure and genetic diversity of *V. pourtalesii* populations as it has been shown in other marine species^90,91^. Direct evidence is, however, lacking as to whether *V. pourtalesii* can reproduce asexually. Populations in this study were characterized by low to moderate levels of genetic diversity and inbreeding, and overall low coancestry between the majority of the samples, which suggests predominantly sexual reproduction.

The genetic connectivity patterns inferred in this study agree with the hydrodynamics of the region, which is largely dominated by two major ocean currents, the Gulf Stream and the Labrador Current^92^. The results from a 3-D biophysical model of *V. pourtalesii* larval dispersal implemented by^47^ broadly matches the genetic connectivity pattern inferred in our study. This match between structural and functional connectivity^93^ is an important result, as for most deep-sea invertebrate species particle-tracking models remain the only tool to assess connectivity, given the paucity of population genetic studies on such taxa (e.g^34,38,94^). , . Structural connectivity models are further compromised by the lack of accurate ocean model products depicting bottom currents and vertical movement, as well as information on spawning time, larval dispersal and behaviour, requiring many simulations to capture all reasonable combinations in the models. Overall, the fast-moving Gulf Stream seems to play a major role in larval dispersal of *V. pourtalesii* along the Atlantic continental shelf of the United States, from the Florida peninsula up to the Carolina Shelf^47^ and this study). Its effect is seen in individuals which were collected further apart (over distances as large as 900 km) but still cluster together genetically. However, the influence of this current weakens with increasing distance and its trajectory, as it turns eastward north of Cape Hatteras, USA, promoting a barrier to gene flow, as suggested by F_ST_ values between northern and southern populations that are an order of magnitude greater than those within these populations. Alongshore population connectivity in the southern population is potentially disrupted when the Gulf Stream changes direction, although Gulf Stream eddies may entrain larvae if they reach the surface, creating potential for a weak permeability in the connections between the southern and northern populations. We note that our sample size for the southern population was small (*N* = 7) and further sampling and analyses (both in the Floridan and Carolina Shelves and in the region between the Florida and Carolina Shelves and the Scotian Shelf) are needed to fully explore the potential for population subdivision in that region. Interestingly, the genetic break in the area near the southern part of the USA has been shown for other deep-sea and shallow organisms^95^. The similar genetic clustering with three defined populations in the Gulf of Mexico, the South-Eastern U.S and the North Atlantic (New England Seamounts and eastern North Atlantic) were shown for the cold-water coral *Lophelia pertusa*, another deep-sea habitat-forming species^86^. Studies on three species of deep-sea cephalopods have shown that there is connectivity between the Gulf of Mexico and the north-western Atlantic Ocean through deep-sea corridors which provide the dispersal through eastern and western basins of the North Atlantic^96^. Though some studies, for example, for the cold-seep mussels from the USA Atlantic margin show high levels of connectivity^97^. All of the above, suggest that influence of the bottom currents might have a high influence on the connectivity of some of the deep-sea species in the region.

The dispersal model determined from genetic data confirmed limited larval exchange between northern and southern populations of *V. pourtalesii* obtained from the functional model by^47^. Structural connectivity modelled results also showed northward unidirectional larval transport between the Straits of Florida and the continental slope south of Cape Hatteras, highly dependent on pelagic larval duration and position in the water column^47^. Simulated larvae released from sites situated north of Cape Hatteras, on the Carolina Shelf, showed low dispersal potential and settled near their source, further supporting the limited long-range connectivity inferred from our study. On the Scotian Shelf, the model by^47^ suggests considerable larval retention with possible unidirectional transport to the Northeast Channel, located west of the populations sampled in this study. Such dispersal patterns would result in high levels of local recruitment on Scotian Shelf *Vazella* grounds consistent with our detection of inbreeding in all populations (Table 1). Larval retention supporting inbreeding is balanced with directed gene flow as indicated by the migrant analyses, creating shallow genetic structure within the northern populations*Vazella pourtalesii* populations showed moderate levels of genetic diversity (*H*_*E*_=0.300) across the studied area, and in all locations, levels of observed heterozygosity were lower than expected, which might be attributed to the inbreeding detected. Although genetic diversity estimates for other deep-sea sponge or coral species are inexistent for the same geographical region, observed values are similar to those found using SNPs in the glass sponge *Aphrocallistes vastus* that forms sponge reefs at similar depths in the Northeast Pacific (*H*_*E*_=0.240–0.323;^74^); but are higher than those observed in two other deep-sea sponges occurring in the Northeast Atlantic, viz. *Phakellia robusta* (*H*_*E*_=0.177^49^) and *Phakellia ventilabrum* (*H*_*E*_=0.131^38^) as well as in a shallow-water species, *Dendrilla antarctica*, from the Antarctic peninsula (*H*_*E*_=0.162^37^). In contrast, genetic diversity observed for *V. pourtalesii* is considerably lower than values observed in several deep-sea and shallow-water sponges studied in other regions, and at various spatial scales, with other markers^35,98^). However, lower levels of expected heterozygosity in SNPs compared to microsatellites have been previously demonstrated^99^.

Low genetic diversity has previously been connected to high levels of inbreeding and population size decline^100^. F_IS_ coefficients, resulting from heterozygosity deficit, were moderate in the whole dataset, although the reported values were significantly lower than in a similar study in the shallow-water demosponge *Dendrilla antarctica* (F_IS_=0.586^37^). It may indicate non-random mating which implies deviations from HWE equilibrium^101^, leading to some cryptic genetic structuring within populations, which in turn might trigger negative effects in population stability. Recent studies suggest that inbreeding plays a significant role in shaping the evolutionary background of marine species as one of the core components of their biology, and is commonly observed among marine invertebrate species^102^. Other population genetics studies, focusing primarily on demosponges in other regions and depths, and using microsatellite markers, also reported high F_IS_ indices between 0.2 and 0.4, rarely exceeding 0.5^99,103^. In general, higher F_IS_ values were associated with shorter distances between populations, higher selection pressures, and lower connectivity reported for the studied sponge populations^32,34^. Our observation fits into a pattern observed throughout the studies of marine invertebrate species with free-spawning and planktonic larvae with a mean F_IS_=0.205^104^.

In addition to larval transport, it is possible that the dispersal of *V pourtalesii* may also be achieved by adults. Turbulent storms in the deep-seabed are common and have shown to be sufficiently strong in the Scotian Shelf to dislodge not only parts but intact adult individuals attached to small rocks or pebbles^43^. However, the extent to which such events may lead to significant transport by bottom currents and contribute to population connectivity was not investigated.

Overall, a deficit of heterozygotes, low to moderate diversity levels together with high positive inbreeding values are quite commonly reported in sponge populations, corals, and marine invertebrates in general^90^. Although asexual reproduction could result in a reduction in genetic diversity and genetic isolation, such explanation seems unlikely based on reasons previously discussed for reproduction strategies in *V.pourtalesii*.

Deep-sea fisheries employing bottom-contacting gears are currently perceived as the most prominent threat to deep-sea vulnerable marine ecosystems globally^105^. This is due to the high sensitivity and low recovery potential of many species conferred by ecological traits such as slow growth rates, high longevity and structural fragility of its dominant species^16,106^. Sponge grounds experience direct and indirect effects of expanding deep-sea fisheries, and particularly in the northwest Atlantic. The Scotian Shelf bioregion is one of richest fishing grounds in the world, covering approximately 476,000 km^2^. Despite being the second smallest among Canada’s Atlantic and Eastern Arctic bioregions, it concentrates about 36% of all the fishing effort in the area (2 million vessel days in total in the period 2005–2014)^80^. The sponge-important benthic areas, including those where high density of *V pourtalesii* occurs, cover approximately 13,000 km^2^ of the Scotian Shelf bioregion. These areas of high associated biodiversity result in a very extensive overlap (77.5%) with the bottom fishing footprint^80^. *V.pourtalesii* has been reported to constitute a considerable part of the bycatch of bottom fisheries in some areas, a large part of which occurs in close proximity of the conservation areas and high-density sponge grounds^40^. From our sample set, 75% were located in areas were bottom trawling occurred at any point between 2005 and 2014. The lowest levels of inbreeding and the highest observed heterozygosity were found in samples from the unfished basin closure, where bottom trawling was prohibited since 2017. No fishing activity was recorded in this area between 2005 and 2014, meaning it has likely remained undisturbed for nearly two decades, or possibly longer, as bottom trawling for pollock in areas with *V.pourtalesii* peaked in the mid-to-late 1980s according to^40^. Moreover, the samples are located further from areas with intensive fishing compared to those in the SCA (Fig. 5). The samples in the SCA that was fished until 2009, showed nearly identical values to samples outside the SCA that are still being fished. This suggests that the absence of fishing over the past 15 years has not significantly impacted genetic diversity. This might be due to the close proximity of the SCA to intensively fished areas (see Fig. 5). The close proximity to locations with high fishing effort, might still affect unfished areas, as is shown by samples outside fished grid cells and outside SCA closures showed the lowest observer heterozygosity, but similar F_IS_ to the SCA and other fished areas. It is therefore, possible that with prolonged absence of fishing disturbance, and larger distance to areas with fishing pressure, inbreeding will reduce.

Considering that climate change is expected to increase the frequency of hurricanes and temperature anomalies in this region, posing additional stresses, fishing on these sponge grounds outside of the conservation areas may be the only threat affecting the sponges that can be mitigated. Future monitoring of the closed areas after sufficient time has elapsed for the populations to regenerate may show increased divergence in genetic diversity between the protected and fished areas. The time frame between the closure of the areas and the collection of the samples for this study was too short (3–9 years) to see an effect of the removal of the fishing disturbance.

The findings in our study suggest that the Emerald Basin and Sambro Bank SCAs, although small in area (1% of the predicted distribution of the species) currently conserve a relatively high proportion of the species genetic diversity at the regional (Scotian Shelf) scale. However, the large number of private alleles found outside of the existing SCAs and the considerable differentiation of the population located in the deeper areas of the Outer Scotian Shelf (Outer Scotian Shelf), suggest that a re-assessment of the boundaries of these SCAs or even the implementation of new SCAs would be beneficial.

This habitat’s fragility on the Scotian Shelf is likely to be further compounded due to the effects of changes in climate conditions predicted for the area in the next decades. Recent research, developing habitat suitability models, predicted a change in distribution of *V. pourtalesii*, with a shift of its populations to deeper water and higher latitudes, under moderate (RCP 4.5) and worst-case (RCP 8.5) CO_2_ emission scenarios. Despite a predicted increase in suitable area under such scenarios, the warming of *V. pourtalesii*’s core habitat (in Emerald Basin) is expected to result in a reduction in its relative likelihood of occurrence in the area, suggesting that present-day *Vazella* grounds will be negatively impacted by climate change^107^. The Northeast Channel, represented by some individuals in the Outer Scotian Shelf population, was identified as a core area of the future distribution of *Vazella*. As this area is already a marine refuge (Northeast Channel Coral Conservation Area), along with the Corsair and Georges Canyons Conservation Area to the south, protection of the genetic diversity represented in the Outer Scotian Shelf population could be achieved through boundary adjustments to accommodate the distribution of *V. pourtalesii* in that area. However, the low number of samples collected there prevented us from assessing in greater detail the diversity and structure of the population in the area, to further inform potential boundary changes.

The patterns of genetic differentiation and connectivity between the different ecoregions and particularly between Florida/Carolina and Scotian Shelf, shows that *V. pourtalesii* does not form a large panmictic population. Therefore, ensuring the protection of the species along its southern range in Canada would be desirable, even if at present dense aggregations of *Vazella* are not known to occur over large spatial extents in these ecoregions, however, there are reports that it is abundant in small aggregations. Despite the low number of individuals collected in the area, those individuals showed strong differentiation, highlighting their uniqueness and importance. This population showed lower genetic diversity, which might have long-term consequences in future, especially, taking into account that recruitment from the large sponge grounds of the Scotian Shelf is limited, making these groups particularly vulnerable both to human activities in the area and natural processes. Similar protective measures should be undertaken in the southern population, potentially in the Blake Plateau region which has already been highlighted as a “Hope Spot” for future conservation attention^108^.

Further, the connectivity shown between the Sambro Bank and Emerald Basin Conservation Areas suggests that they are part of an ecological network consistent with expectations for OECMs^22^. Although connectivity was greater in one direction than the other, bi-direction gene flow was observed through the migrant analyses. This suggests that the two areas are mutually supportive of one another. Although there were insufficient data to analyse gene flow from these areas to the Northeast Channel Conservation Area, structural connectivity models suggest that this connection is likely with two week pelagic larval duration^47^. Thus, any *Vazella* occurring in that closure would also be a part of the ecological network protected by regional OECMs.

## Conclusion

In summary, we found a high degree of genetic connectivity between populations with a minimal genetic structure across the Scotian Shelf. The conservation areas, while maintaining a similar level of genetic diversity to the samples from outside the protected zones, revealed only two unique alleles not found outside these areas. In contrast, 23 unique alleles were recovered exclusively from areas outside the protective zones. This suggests that while closures may preserve similar levels of genetic diversity, a significant number of alleles—and potentially important evolutionary potential—could be lost if connectivity between sponge grounds and surrounding areas is limited. Moreover, the genetic connectivity observed in this study may not reflect contemporary gene flow but rather remnants of historical gene flow, further, the number of effective migrants might be too low to maintain high levels of heterozygosity^109,110^. Insufficient time may have passed to observe the resulting genetic consequences. Overall, lower observed heterozygosity compared expected heterozygosity, might reflect this. Furthermore, the lack of fishing, and distance to intensive bottom trawling seems to have a positive effect on the inbreeding, as compared samples in fished and close to fishing areas. In light of our study’s findings, we recommend that protected areas for *V pourtalesii* be better connected and situated farther from active fishing areas to ensure the resilience of these populations in the future. We highlight the need for both a precautionary and adaptive approach to preserve the vulnerable marine ecosystem and the goods and services it provides. Adaptive approach is the key to make sure sponge conservation areas are tracking potential range shifts under climate change, and monitoring genetic changes in the future as recommended in^111^. It is also important to articulate conservation policy at an international level since sponges are connected across borders.

## Electronic supplementary material

Below is the link to the electronic supplementary material.

Supplementary Material 1.

## Data Availability

[Note – the data is under embargo until accepted for publication] RADseq data is available at the Short Read Archive (SRA accession: PRJNA1075922, and SRR27950950:27951029). Additionally, the VCF file containing the filtered SNPs can be accessed on Figshare (10.6084/m9.figshare.25215281).
