# Supplementary material for: Population genomics and connectivity of Vazella pourtalesii sponge grounds of the northwest Atlantic with conservation implications of deep sea vulnerable marine ecosystems
Source: Sci Rep. 2025 Jan 9;15:1540. doi: 10.1038/s41598-024-82462-z (PMC11718047; doi:10.1038/s41598-024-82462-z)
Supplement: Supplementary file 1 — Supplementary Material 1. [file 41598_2024_82462_MOESM1_ESM.pdf]

Supplementary Information to:

Population genomics and connectivity of *Vazella pourtalesii* sponge grounds of the northwest Atlantic with conservation implications of deep sea Vulnerable Marine Ecosystems

Anna Patova[1*], Pedro A. Ribeiro[1], Francisco J. Murillo[2], Ana Riesgo[3,4], Sergi Taboada[3,4,5,6], Shirley A. Pomponi[8], Hans Tore Rapp[1,I], Ellen Kenchington[2], Joana R Xavier[7,1]

1 Department of Biological Sciences, University of Bergen, Bergen, Norway
2 Fisheries and Oceans Canada, Bedford Institute of Oceanography, Dartmouth, Nova Scotia, Canada, B2Y 4A2
3 Departamento de Biodiversidad y Biología Evolutiva, Museo Nacional de Ciencias Naturales, Consejo Superior de Investigaciones Científicas, Calle de José Gutiérrez Abascal, Madrid, Spain
4 Life Sciences Department, The Natural History Museum, Cromwell Road, London SW7 5BD, UK
5 Departamento de Biodiversidad, Ecología y Evolución, Universidad Complutense de Madrid, Facultad de Ciencias, 28049, Madrid, Spain
6 Departamento de Ciencias de la Vida, EU-US Marine Biodiversity Group, Universidad de Alcalá, 28871 Alcalá de Henares, Spain.
7 CIIMAR – Interdisciplinary Centre of Marine and Environmental Research of the University of Porto, 4450-208 Matosinhos, Portugal
8 Harbor Branch Oceanographic Institute, Florida Atlantic University, Fort Pierce, Florida, 34946, USA

[I] deceased
* Corresponding author: Anna Patova

Table S1: Collection data of the samples analyzed in this study in each sampling area. Stations marked with * are located inside a Sponge Conservation Area. For additional information see Supplementary Material 1.

Sampling area (code)	Station	Lat. (N)	Long. (W)	Depth (m)	Gear	N
Scotian Shelf						
Emerald Basin (EMB)	HUDSON16019*	44,319	-62,605	199	ROV	13
	NED2017020195	44,180	-62,388	134	TRL	3
Sambro Bank (SBB)	MLB2017_001004*	43,895	-63,077	154	ROV	5
	MLB2017_001005*	43,894	-63,076	160	ROV	1
	MLB2017_001006*	43,894	-63,076	161	ROV	2
	MLB2017_001020*	43,875	-63,055	186	ROV	5
	NED2017020013	43,982	-63,743	183	TRL	16
	NED2017020194	44,157	-63,118	170	TRL	5
	NED2017020074	43,998	-63,183	176	TRL	1
	NED2017020075	43,998	-63,183	176	TRL	1
Outer Scotian Shelf (OSS)	NED2017020007	43,192	-63,170	136	TRL	5
	NED2017020006	43,307	-63,243	156	TRL	3
	NED2017020005	43,443	-63,557	179	TRL	2
	NED2017020073	42,725	-67,489	197	TRL	1
	NED2019030111	42,873	-63,570	170	TRL	4
	NED2019030021	42,184	-65,873	238	TRL	1
	NED2019030130	43,339	-63,215	174	TRL	1
	NED2019030132	42,828	-62,816	349	TRL	2
	NED2019030131	42,839	-63,056	137	TRL	2
Florida and Carolina Shelves (FCS)						
	MBRC 7-VI-06-1-001	26,125	-79,844	297	SUB	1
	MBRC 26-V-04-2-001	26,021	-79,821	430	SUB	1
	MBRC 27-VIII-02-1-001	31,702	-79,127	519	SUB	1
	MBRC 14-VII-10-1-009	24,251	-81,789	314	SUB	1
	MBRC 6-VIII-10-1-001	24,252	-84,791	380	SUB	1
	MBRC 7-VI-07-2-002	24,236	-81,599	323	SUB	1
	EX1806_D10	33,570	-76,470	374	ROV	1

Table S2: The values for migration patterns, evaluating A) Jost's D values B) "GST" or Nei's Gst

Comparison	Nei's G_{ST}	Jost D	N_m
Emerald Basin –> Sambro Bank	1.000	1.000	1.000
Sambro Bank -> Emerald Basin	0.685	0.640	0.680
Emerald Basin –> South of Emerald The Outer Scotian Shelf	0.540	0.516	0.540
South of Emerald The Outer Scotian Shelf –> Emerald Basin	0.410	0.489	0.410
Emerald Basin –> Florida/Carolina	0.093	0.153	0.090
Florida/Carolina -> Emerald Basin	0.199	0.289	0.190
Sambro Bank -> South of Emerald The Outer Scotian Shelf basin	0.742	0.534	0.740
South of Emerald The Outer Scotian Shelf Basin -> Sambro Bank	0.809	0.641	0.810
Sambro Bank -> Florida/Carolina	0.102	0.121	0.100
Florida/Carolina -> Sambro Bank	0.269	0.325	0.270
South of Emerald The Outer Scotian Shelf Basin -> Florida/Carolina	0.106	0.113	0.240
Florida/Carolina -> South of Emerald The Outer Scotian Shelf Basin	0.273	0.333	0.110

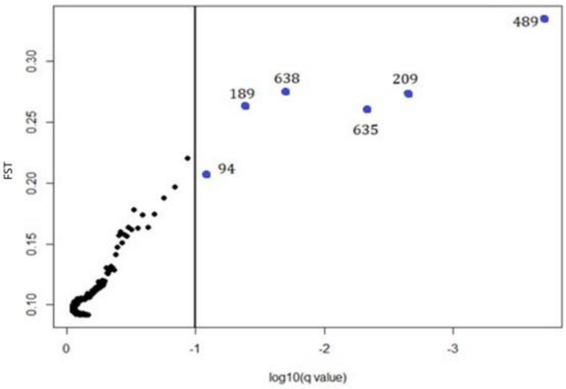

Figure S1: Graphical representation of outlier detection SNPs in *Vazella pourtalesii* dataset using BayeScan using 1,206 candidate SNPs. The SNP-specific F_{ST} coefficient (locus-specific genetic divergence among populations) is plotted against the log10 (q-value, a decision factor in logarithmic scale (base 10) to determine selection) with the vertical line indicating Bayes Factor (log10)> 1.5. Blue dots show SNPs detected as putative outliers (*q*-value<0.05).

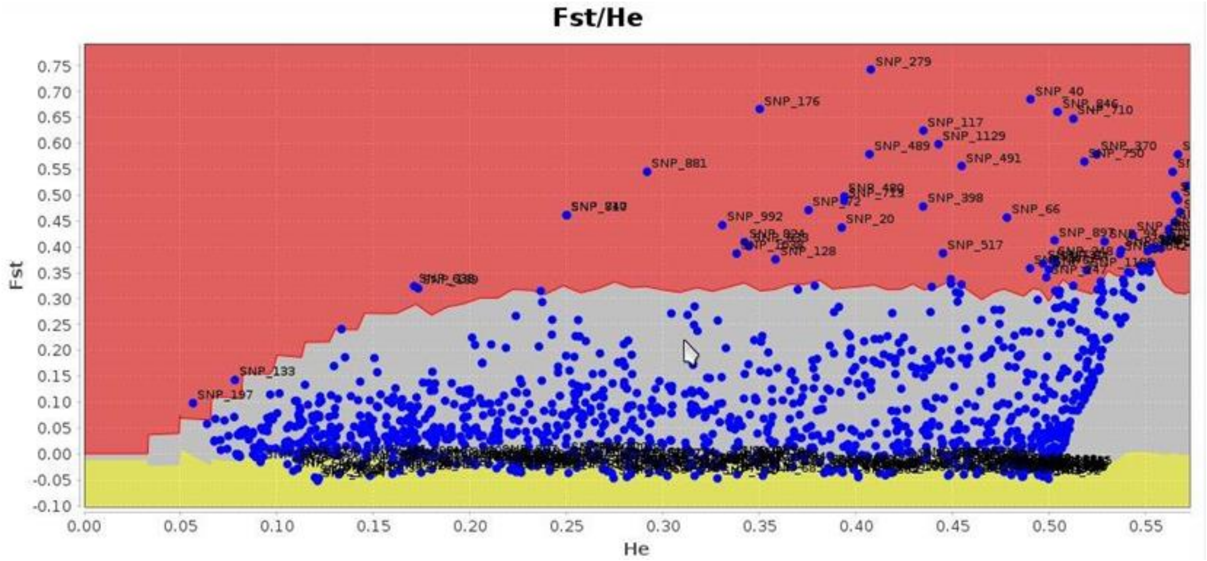

Figure S2: FST outliers detected by LOSITAN in groups. Red area – candidates for positive selection, yellow area – candidates for balancing selection, grey area – neutral SNPs.

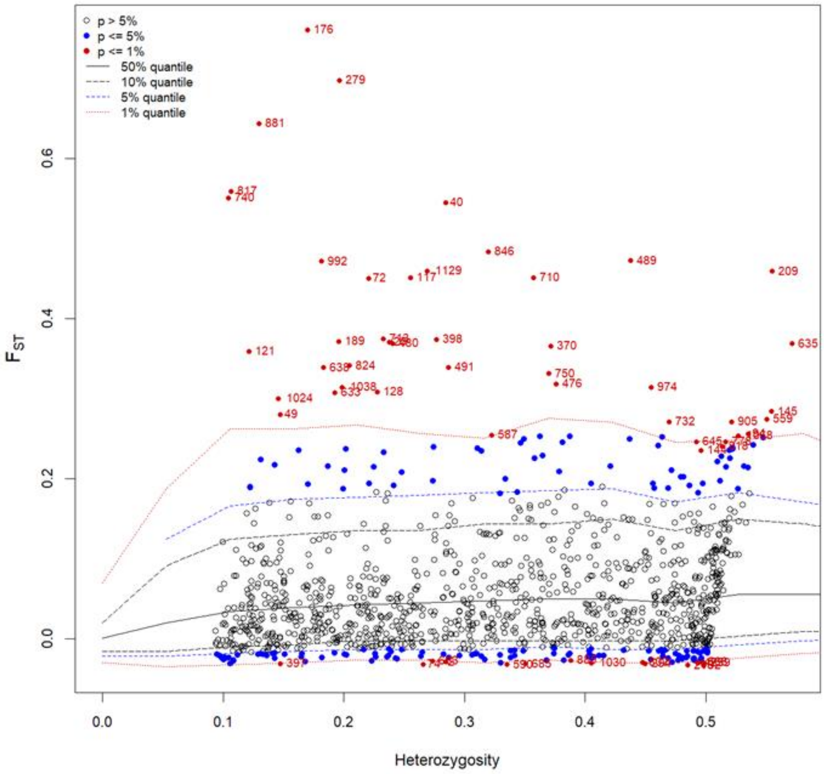

Figure S3: FST outliers detected by Arlequin in groups. SNP Loci in red are above the 1% quantile, and blue above 5% quantile. The loci above 1% quantile were removed. .

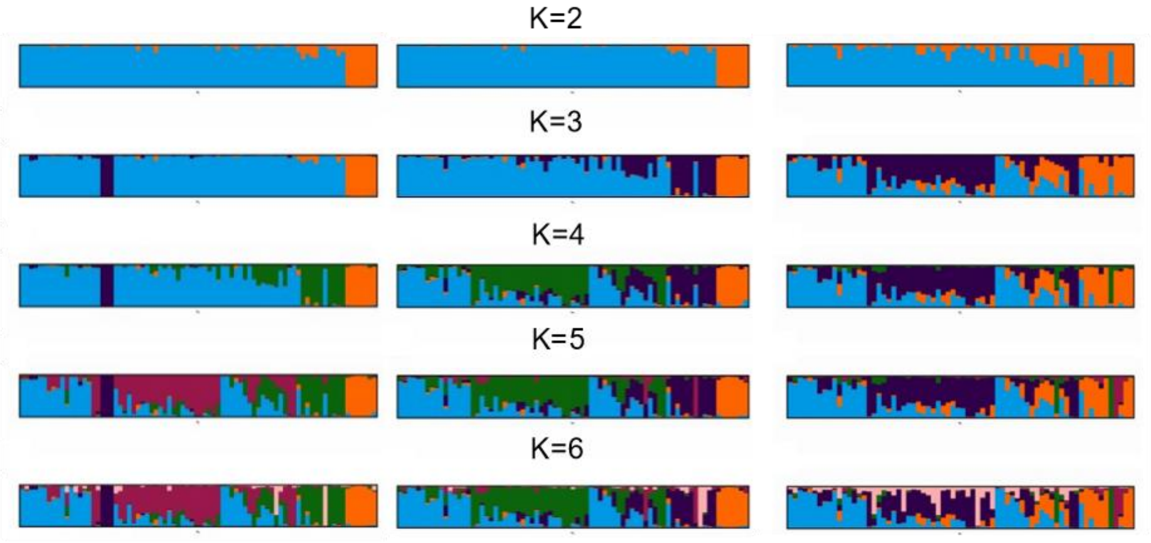

Figure S4: STRUCTURE results for K=2 to K=6 with 1,102 neutral SNPs for (first column) 80 individuals, all successfully sequenced individuals (second column) 77 Individuals (excluding 3 individuals from Sambro Bank) (thirth column) 70 individuals from the Scotian Shelf.
